# Reduced aggression and foraging efficiency of invasive signal crayfish (*Pacifastacus leniusculus*) infested with non-native branchiobdellidans (Annelida: Clitellata)

**DOI:** 10.1186/s13071-015-1199-1

**Published:** 2015-11-17

**Authors:** J. James, K. E. Davidson, G. Richardson, C. Grimstead, J. Cable

**Affiliations:** School of Biosciences, Cardiff University, Cardiff, CF10 3AX UK; Natural Resources Wales, Rivers House, St. Mellons, Cardiff, CF3 0EY UK

**Keywords:** Freshwater invasions, Crustacean parasites, Intraspecific competition, Co-introduced symbionts, *Xironogiton victoriensis*

## Abstract

**Background:**

Biological invasions are a principal threat to global biodiversity and identifying the determinants of non-native species’ success is a conservation priority. Through their ability to regulate host populations, parasites are increasingly considered as important in determining the outcome of species’ invasions. Here, we present novel evidence that the common crayfish ecto-symbiont, *Xironogiton victoriensis* (Annelida: Clitellata) can affect the behaviour of a widespread and ecologically important invader, the signal crayfish (*Pacifastacus leniusculus*).

**Methods:**

To assess the signal crayfish–*X. victoriensis* relationship naïve crayfish were infested with an intensity of worms typically observed under natural conditions. Over a 10-week period the growth rate and survivorship of these animals was monitored and compared to those of uninfested counterparts. Complementary dyadic competition and foraging experiments were run to assess the behaviour of infested compared to uninfested animals. These data were analysed using General Linear Models and Generalized Linear Mixed Models.

**Results:**

Whilst *X. victoriensis* did not affect the growth rate or survivorship of signal crayfish under laboratory conditions, infested animals were significantly less aggressive and poorer foragers than uninfested individuals.

**Conclusions:**

Through reducing aggression and foraging efficiency, infestation with *X. victoriensis* may disrupt the social structure, and potentially growth rate and/or dispersal of afflicted crayfish populations, with potential effects on their invasion dynamics. This is important given the widespread invasive range of crayfish and their functional roles as ecosystem engineers and keystone species.

## Background

Biological invasions are a principal threat to global biodiversity [1], and freshwater ecosystems are particularly vulnerable to the effects of invasive species [2]. These threats are likely to intensify with the predicted increase in future invasion rates [3, 4]. Of all introduced species, around 1 % will ultimately become invasive [5, 6], and identifying the determinants of this is a conservation priority. Parasites, or the lack thereof, can alter host invasion dynamics [7–9] and in some cases are considered to be the key factor determining the outcome of species’ invasions [10].

Most often the role of parasites in determining invasion success is considered in the context of the Enemy Release Hypothesis which postulates that escape from natural enemies facilitates the establishment and spread of non-native species [11]. In their non-native range, introduced animals can escape over 75 % of their native parasites [7], only about 25 % of which are replaced by parasites acquired from the recipient ecosystem [12]. It is unsurprising, therefore, that the role of many parasites (co-introduced or acquired from their new habitat) in controlling invaders is comparatively understudied [13, 14]. The effects of parasites on non-native hosts may be equally as profound as those resulting from parasite absence. For instance populations of invasive rusty crayfish (*Orconectes rusticus*) in North America exist in alternate abundance states that can be at least partially explained by the presence of a trematode parasite, *Microphallus* sp., which reduces crayfish abundance and population growth [14].

Identifying factors that allow non-natives to thrive in new environments is particularly important for crustaceans, which comprise a disproportionately large proportion of the 13 freshwater species listed among the 100 ‘worst’ invasive species [15]. In particular crayfish have been widely translocated for aquaculture [15, 16], and their invasive range now extends throughout most of Europe [17, 18] and into Asia [19]. Invasive crayfish pose a significant threat to freshwater biodiversity and ecosystem functioning [20, 21]. They are host to a wide range of fungi, viruses, bacteria, protists and metazoans [22], many of which may alter their invasion success if co-introduced. It is well established that the spread of North American crayfish across Europe is facilitated by transmission of *Aphanomyces astaci*, the causative agent of crayfish plague, to susceptible European crayfish, in which infection is typically lethal [23–26]. In contrast, North American crayfish species are largely resistant to the disease [23] and therefore gain a competitive advantage over native crayfish species.

Whilst *A. astaci* has been extensively reported, other lesser-known groups of symbionts may also affect crayfish invasion dynamics. One such group are the branchiobdellidans, ecto-symbiotic annelids that have a widespread global distribution across the Nearctic and two disjunct regions of the Palearctic [27]. Invasive American branchiobdellidans were first recorded in Europe on North American signal crayfish (*Pacifastacus leniusculus*) from Sweden during the 1960s [28] and have since been found in Austria [29], Finland [30], Spain [27, 31, 32], Italy [33, 34], France [29, 35, 36], Hungary [37], and most recently from the UK [38]. The impact of branchiobdellidans on the invasion dynamics of crayfish in these countries is however difficult to predict given the variable nature of the crayfish-branchiobdellidan relationship [39]. Although branchiobdellidans are generally considered commensals [40–42], their association with crayfish can vary from mutualism [43–45] to parasitism [44, 46, 47] depending on the host, branchiobdellidan species and density, and environmental conditions. Also, many species of branchiobdellidans have been categorized as commensals based only on crayfish growth rate and/or survivorship studies (e.g., [41]), although it is known that ecto-symbionts alter host behaviour in multiple ways, some of which reduce host fitness (e.g., [48]). Therefore whilst branchiobdellidans clearly have the potential to influence the invasion dynamics of non-native crayfish; elucidating the nature of this effect is complex.

Here, in a series of laboratory experiments, we assessed the impact of *Xironogiton victoriensis* (Annelida: Clitellata) on the growth rate, survivorship and behaviour of their native signal crayfish hosts [49]. Our aim was to investigate how these symbionts may influence the invasion dynamics of signal crayfish in their non-native range.

## Methods

### Collection and husbandry of experimental animals

In June 2013, *Xironogiton victoriensis* infested signal crayfish were collected from the River Gavenny (Abergavenny, Wales) and uninfested crayfish from the Bachowey River (Powys, Wales). All crayfish were harvested using standardised manual searches (stone turning and kick sampling). Following capture, animals from each population were transported to Cardiff University and housed in separate 100 L tanks filled with dechlorinated water (15 ± 1 °C) under a 16 h: 8 h light/dark regime, at a density of ca. 15 individuals/m^2^. All experiments were conducted under these environmental conditions, and using only crayfish from the uninfested population. Stock tanks were supplied with gravel substrate (2 cm) and sufficient refuges (plastic tubes and plant pots) for all animals. Crayfish were fed daily with Tetra Crusta crayfish food pellets and 50 % water changes were performed weekly. Crayfish with regenerating or missing chela or displaying signs of disease were not used in any experiment. Upon termination of experiments, all animals were humanely destroyed by freezing at −20 °C, in accordance with the Wildlife and Countryside Act, 1981.

For use in foraging efficiency trials, *Gammarus pulex* were collected from the same location as the uninfested crayfish, Bachowey River (Powys, Wales), in May 2014 using a fine-mesh dip net. These gammarids were maintained in a 60 L tank filled with dechlorinated water and housed under the same temperature and lighting conditions as the experimental crayfish. Gammarids were fed daily with a mixture of *Spirulina*, yeast and dechlorinated water.

### Experimental infestations with branchiobdellidans

Worms carefully dislodged from naturally infested signal crayfish using the edge of blunt forceps into a Petri dish, were checked that they remained active and undamaged using a dissecting microscope with fibre optic illumination. Worms in good condition were then transferred on to the carapace of recipient animals using forceps, and observed to ensure that they had fully attached to the recipient crayfish. Experimental infestation loads were based on those in a naturally infested wild population of signal crayfish, which varied according to host size ([38] and see below).

### The effect of branchiobdellidan infestation on signal crayfish growth and behaviour

To investigate the effects of long term exposure to branchiobdellidans on crayfish growth, individually maintained animals were weighed weekly over a 10 week period (*n* = 40 per treatment, sex and size matched to within 10 % carapace length, CL), and then interactions between infested vs uninfested individuals were assessed over one day. Crayfish were housed in 15 L tanks containing a single plant pot refuge and fed every 48 h with 2 g of commercial fish food flakes. Crayfish in the infested treatment were grouped in the following size categories (CL, mm): 28–31; 32–35; 36–39; 40–43; 44–48, and infected with 21, 28, 65, 101 and 154 worms respectively, these reflect natural burdens of *X. victoriensis* on signal crayfish [38]. Weekly, infested crayfish were screened and, if their branchiobdellidan burden declined at any point, new worms were added to maintain a constant infection intensity on each crayfish throughout the experiment. Branchiobdellidan declines were expected as crayfish commonly regulate worm densities through grooming [50], a behaviour frequently observed during our experiments. If a crayfish moulted, the moult was left in the tank for at least 24 h to allow worms to transfer back onto the crayfish.

At the end of the 10 week experiment, dyadic competition trials were conducted between infested and uninfested crayfish in an experimental tank (L60 cm x W30 cm x D30 cm) separated into three compartments using a mobilised plastic divider. Prior to trials commencing, an infested crayfish, and a sex and size matched (within 10 % carapace length) uninfested crayfish, were placed on either side of the divider. After 5 min acclimatisation the mobile dividers were lifted and interactions between the infested and uninfested crayfish recorded for 1 h using Micropix USB webcam cameras. The number of intraspecific interactions made by each crayfish during the trial period was subsequently recorded. It was not possible to distinguish which crayfish were infested in these webcam recordings, thus all observations were made “blind” using an identifying nail polish mark applied to the dorsal carapace to recognise individuals. The four types of intraspecific behaviours recorded were characterised as: i) fight - whereby a physical interaction is initiated (chelae strike/locking), ii) threat - where one crayfish approaches another in a threatening posture (e.g., chelae raised) but no physical contact is made, iii) retreat - where a crayfish retreats from a physical interaction (i.e., backs down from a fight) and, iv) avoid - when a crayfish moves away from an approaching crayfish but no physical interactions have taken place (i.e., the crayfish moves away from a threatening opponent). For these competition experiments, we recorded the number of worms on the infested and uninfested crayfish at the start and end of the trial respectively and the total contact duration(s) between the pair over the 1 h test period.

### The effect of branchiobdellidan infestation on crayfish foraging efficiency

To determine whether short-term exposure of naïve signal crayfish to branchiobdellidans altered their foraging efficiency we experimentally infested signal crayfish (*n* = 25) with *X. victoriensis* and assessed their predation on gammarids, compared to uninfested controls (*n* = 25). Crayfish were housed individually in 10 L tanks, containing a single plant pot refuge, and allowed to acclimatise for three days. On Day 3 half of the crayfish were infected with branchiobdellidans and the other half sham infected by handling alone without exposure to *X. victoriensis*. Infested crayfish received 90 worms, reflecting the mean natural infection intensity for crayfish of the size used in the experiment, 38.6–62.1 mm carapace length [38]. Following experimental infection, each crayfish was returned to its respective 10 L tank and left to acclimatise, without being fed, for 3 days prior to foraging experiments commencing. On Day 6, the refuge was removed from all tanks and five live gammarids (size range: 6–12 mm body length) were introduced. Latency to attack (time taken to launch the first attack, irrespective of success) and the number of gammarids each crayfish consumed was recorded at 10, 30, 60 min and 18 h. At the end of the experiment, the number of worms remaining on each crayfish was also recorded.

### Statistical analysis

All analyses were conducted in the R statistical package v2.15.1 [51] with Generalized Linear Mixed Models (GLMMs) being conducted using the ASReml-R (version 3.0 package within the R interface). For each model the error distribution (quasi-poisson, gaussian, poisson or Gamma) was selected by; visualizing histograms of the dependent variable, assessing residual plots as recommended by Pinheiro and Bates [52] and, specifically for quasi-poisson models, measuring over-dispersion using the dispersion parameter, theta [53]. Non-significant terms were sequentially deleted from starting models using Analysis of Variance for General(ised) Linear Models [54] and the Wald statistic for GLMMs [53], and only significant terms are reported. The fit of the refined models, was assessed using residual plots [52].

A General Linear Model with a gaussian error distribution and identity link function was used to assess whether the percentage change in weight of crayfish over the experiment was significantly different between infested (*n* = 36) and uninfested crayfish (*n* = 26 at the end of the experiment). Crayfish size (carapace length, CL mm), sex and whether or not the crayfish moulted during the experiment were included as independent variables, as well as interaction terms between infestation status and both crayfish size and moult status. A Chi square test was used to compare the number of crayfish moults in the infected (*n* = 36) and uninfested group (*n* = 26).

Generalised Linear Models with a quasi-poisson error distribution and log link function were used to assess the effect of crayfish infestation status (branchiobdellidan infested or control), sex and size (CL mm) on their behaviour. Data for each behaviour type (i.e., fight, threat, retreat, avoid) were analysed independently and models also included the total number of all behaviours performed by each crayfish as a controlling variable.

For crayfish in the infested treatment group (*n* = 28) quasi-poisson Generalised Linear Models (log link function) were run to assess the impact of infestation intensity (measured as the number of worms on the infested crayfish at the start of the trial) on crayfish behaviour. As infestation intensity and crayfish size (CL) were positively correlated (Pearson’s correlation: *t* = 6.02, *df* = 26, *P* < 0.001) analysing them as separate independent variables could cause issues relating to collinearity (see [53]). Therefore, for each behaviour, we ran three separate GLMs; one including crayfish size (CL) and infestation intensity as independent variables, one just including crayfish size (CL), and another just including infestation intensity. All models also included as independent variables, crayfish sex and the total number of all behaviours performed by each crayfish. All behaviour types were analysed individually. A separate Generalised Linear Model with a Gamma error distribution and a log link function was used to assess how crayfish infestation intensity (i.e., the number of worms on the infested crayfish at the start of the trial) is influenced by sex and size (CL mm).

A Kendall-Tau correlation was used to determine if the number of worms transmitted to the originally uninfested crayfish was correlated to the number of worms on the infested crayfish at the start of the trial. We also used a Kendall-Tau correlation to test whether the proportion of worms on the infested crayfish that were transmitted to the originally uninfested animal over the 1 h trial period was correlated with their total contact duration(s).

A General Linear Model with a gaussian error distribution and identity link function was used to investigate the effect of infestation status (control, *n* = 25, or infected, *n* = 25), crayfish size (carapace length) and crayfish sex on the (log transformed) latency to attack gammarid prey. A GLMM with a gaussian error structure and identity link function was used to investigate the effect of infestation status, crayfish size and crayfish sex on the number of gammarids captured over the duration of the experiment. For crayfish in the infested treatment group (*n* = 25), GLMMs (gaussian family, identity link) were performed to assess the effect of infestation intensity (at the end of the trial) on the number of gammarids captured. For this, three separate GLMMs were run because of the collinearity between crayfish size and infestation intensity (Pearson’s correlation: *t* = 2.55, *df* = 23, *P* = 0.02); one including infestation intensity, crayfish size and crayfish sex as independent variables, the second including just infestation intensity and sex, and the third including just crayfish size and sex. To control for repeated measures, both crayfish identification number and time of record (10, 30, 60 min or 18 h) were included as random effects in all GLMMs. Interactions between all independent variables were included in each initial model.

## Results

### The effect of branchiobdellidan infestation on signal crayfish growth and behaviour

Over the 10 week experiment, there was no significant difference in the percentage weight change (GLM: *P* > 0.05) or number of moults (X^2^ = 0.008, *df *= 1, *P* = 0.98) between uninfested and infested signal crayfish. Smaller crayfish and those that moulted gained more weight than larger crayfish or those that did not moult (GLM: F_1,63_= 97.38, *P* < 0.0001, F_1,63_= 16.78, *P* < 0.001 respectively). There was no apparent difference in growth between male and female crayfish (*P* > 0.05).

**Fig. 1:**
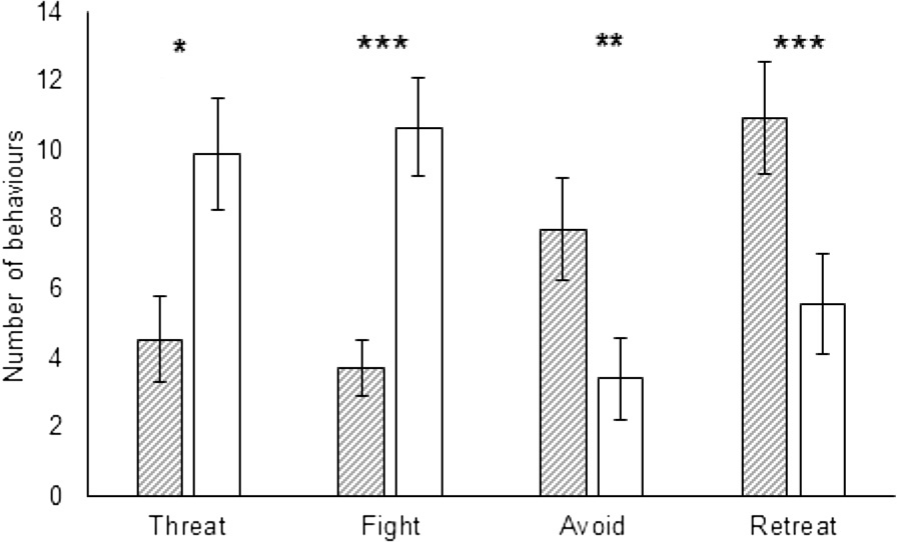
Effect of branchiobdellidan infestation on host aggressive behaviour. Mean (±SE) number of threat, fight, avoid and retreat behaviours performed by *Xironogiton victoriensis* infested (hatched bars) and uninfested (white bars) signal crayfish in dyadic competition experiments. * *P* < 0.05, ** *P* < 0.01, *** *P* < 0.0001

During dyadic interactions, infested crayfish performed significantly less fight (GLM: Deviance_1, 53_ = 313.42, *P* < 0.0001) and threat (Deviance_1, 53_ = 405.46, *P* = 0.02) behaviours, and significantly more retreat (Deviance_1, 53_ = 349.35, *P* < 0.0001) and avoid (Deviance_1, 53_ = 445.90, *P* < 0.01) behaviours than uninfested crayfish (Fig. 1). No effects of sex or size on crayfish behaviour were detected (*P* > 0.05 for all).

For infested crayfish the number of avoid behaviours performed by females was significantly higher than that of male crayfish (*P* < 0.05) for all three models (i.e., including crayfish size and infestation intensity as variables together and singularly, Table 1). Males performed more threat behaviours than female crayfish (GLM: Deviance_1,25_ = 200.31, *P* = 0.05) but this effect was only significant when not controlling for infestation intensity (Table 1). (GLM: LRT_1,25_ = 6.31, *P* = 0.01; LRT_1,25_ = 4.70, *P* = 0.03, respectively). Infestation intensity was negatively correlated with the number of crayfish avoid behaviours (Deviance_1,24_ = 116.20, *P* = 0.05), although this was only significant when not controlling for crayfish size (CL) (Table 1). Infestation intensity was positively correlated with the number of threat behaviours initiated by crayfish (*P* < 0.01) when infestation intensity was included in the two models with and without crayfish size as a variable (Table 1). The number of worms on infested crayfish at the start of the trial was positively correlated with crayfish size (GLM: F_1,25_ = 77.98, *P* < 0.001) and higher for male than female crayfish (F_1, 25_ = 18.30, *P* < 0.001).

**Table 1:** The structure of Generalized Linear Models used to investigate the effects of sex, size (carapace length, CL mm) and infestation intensity on the number of avoiding, retreating, threatening and fighting behaviours performed by *Xironogiton victoriensis* (Annelida: Clitellata) infested signal crayfish (*Pacifastacus leniusculus*)

Dependent variable	Independent variables	Significant terms	Deviance	Df	*P*
No. of avoid behaviours	Sex, Size (CL mm), Infestation Intensity, Total Behaviours	Sex	129.54	1,24	<0.01
		Size (CL mm)	116.20	1,24	0.03
		Total Behaviours	212.15	1,24	<0.0001
	Sex, Size (CL mm), Total Behaviours	Sex	129.54	1,24	<0.01
		Size (CL mm)	116.20	1,24	0.03
		Total Behaviours	212.15	1,24	<0.0001
	Sex, Infestation Intensity, Total Behaviours	Sex	119.80	1,24	0.03
		Infestation Intensity	116.20	1,24	0.05
		Total Behaviours	195.88	1,24	<0.0001
No. of retreat behaviours	Sex, Size (CL mm), Infestation Intensity, Total Behaviours	Total Behaviours	219.89	1,26	<0.0001
	Sex, Size (CL mm), Total Behaviours	Total Behaviours	219.89	1,26	<0.0001
	Sex, Infestation Intensity, Total Behaviours	Total Behaviours	219.89	1,26	<0.0001
No. of threat behaviours	Sex, Size (CL mm), Infestation Intensity, Total Behaviours	Infestation Intensity	200.31	1,25	<0.01
	Sex, Size (CL mm), Total Behaviours	Sex	200.31	1,25	0.05
	Sex, Infestation Intensity, Total Behaviours	Infestation Intensity	200.31	1,25	<0.01
No. of fight behaviours	Sex, Size (CL mm), Infestation Intensity, Total Behaviours	Nothing	N/A	N/A	N/A
	Sex, Size (CL mm), Total Behaviours	Nothing	N/A	N/A	N/A
	Sex, Infestation Intensity, Total Behaviours	Nothing	N/A	N/A	N/A

For significant terms (*P* ≤ 0.05) the deviance, degrees of freedom (df) and *P*-values are reported. In each model, the total number of all types of behaviours performed by crayfish was also included as a controlling variable, which was retained in the final refined models (after stepwise deletions of non-significant terms based on Analysis of Variance) even if not significant

**Fig. 2:**
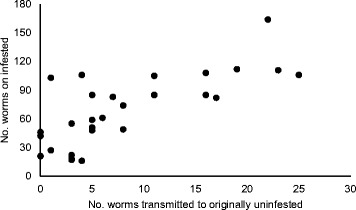
Correlation between host branchiobdellidan infestation intensity and transmission potential. Number of branchiobdellidans on the infested crayfish in relation to the number of worms transmitted to the originally uninfested animal during dyadic competition experiments

In behavioural trials at least one worm was successfully transmitted to 89.3 % of the originally uninfested hosts within one hour. The total number of worms transmitted to the uninfested animal was positively correlated to the number of worms on the infested individual at the start of the trial (Kendall-Tau correlation test: *z* = 4.09, *P* < 0.001) (Fig. 2). There was, however, no significant correlation between the proportion of worms on the infested crayfish that were transmitted to the originally uninfested animal and their total contact duration (Kendall-Tau correlation: *z* = 1.36, *P* = 0.17).

### The effect of branchiobdellidan infestation on crayfish foraging efficiency

**Fig. 3:**
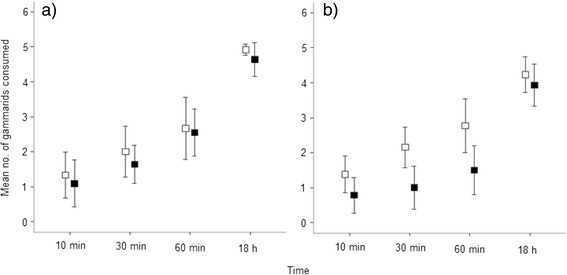
Effect of branchiobdellidan infestation on host foraging efficiency. Mean number of gammarids consumed (±95 % CI) by uninfested (unfilled boxes) and *Xironogiton victoriensis* infested (filled boxes) female (**a**) and male (**b**) signal crayfish after 10, 30, 60 min and 18 h

Infested crayfish captured fewer gammarids than uninfested crayfish at each time point, and this difference was significant overall (F_1,197_ = 12.76, *P* < 0.001; Fig. 3), although there was no difference in the latency to attack between these control and treatment groups (*P* > 0.05). Within the infested group (*n* = 25), crayfish with a higher infestation intensity captured fewer gammarids (Table 2). Infestation with *Xironogiton victoriensis* is predicted to reduce prey consumption by 19.6 % for female and 22.6 % for male crayfish (GLMM). Irrespective of infestation status male crayfish consumed fewer gammarids than female crayfish, with crayfish sex a significant term in both the infestation status (F_1, 197_ = 5.80, *P* = 0.017) and infestation intensity (Table 2) models (Fig. 3).

**Table 2:** The structure of Generalized Linear Mixed Models used to investigate the effects of sex, size (carapace length, CL mm) and infestation intensity on the number of gammarids consumed by *Xironogiton victoriensis* (Annelida: Clitellata) infested signal crayfish (*Pacifastacus leniusculus*)

Fixed terms	Random terms	Significant fixed terms	F (incremental)	Df	*P*
Sex, Size (CL mm), Infestation Intensity, Size: Sex, Sex: Infestation Intensity, Size: Infestation Intensity	Time in experiment, Crayfish ID.	Sex	4.84	1,94	<0.01
		Infestation Intensity	15.83	1,94	0.03
Sex, Size (CL mm), Size: Sex	Time in experiment, Crayfish ID.	Sex	9.38	1,95	<0.01
Infestation Intensity, Sex, Sex: Infestation Intensity	Time in experiment, Crayfish ID.	Sex	10.30	1,94	0.03
		Infestation Intensity	10.36	1,94	<0.01

For significant terms (*P* ≤ 0.05) the F-statistic, degrees of freedom (df) and *P*-values are reported

## Discussion

Here, we find that whilst the branchiobdellidan *Xironogiton victoriensis* did not affect the growth rate of invasive signal crayfish (*Pacifastacus leniusculus*), infested animals were less aggressive and less efficient foragers than their uninfested counterparts. These behavioural effects may reduce the overall fitness of infested crayfish, in which case *X. victoriensis* would be considered parasitic on signal crayfish. Field studies are, however, needed to assess if the observed behavioural changes of *X. victoriensis*-infested signal crayfish translate into fitness costs in the wild. Regardless, the current study demonstrates that branchiobdellidans can alter host behaviour in multiple ways, thus determining the nature of crayfish-branchiobdellidan relationships is not straightforward.

Branchiobdellidans have variable effects on crayfish growth depending on worm species, density and environmental conditions [41, 43–45]. Gill frequenting branchiobdellidans, such as *Branchiobdella kobayoshi* and some *Cambarincola* species', clean epibionts from the branchial chambers promoting host respiration and growth [43–45]. This cleaning behaviour may, however, only be beneficial towards crayfish under conditions of high environmental fouling pressure [45]. Indeed, when worm densities exceed epibiont availability, branchiobdellidans may switch to a diet of host gill tissue [44]. There is some evidence that high densities of gill frequenting branchiobdellidans may reduce host growth rate [44]. As *X. victoriensis* is not known to occupy crayfish gill chambers it is perhaps unsurprising that we did not detect any effects of infestation on host growth rate. A study using *Cambarincola fallax*, which primarily inhabits the subrostral region of the crayfish exoskeleton [55], also failed to detect any effects of infestation on host growth rate [41].

The effect of branchiobdellidans on the agonistic behaviour of their crayfish hosts has, to our knowledge, never previously been assessed. Overall, we found that branchiobdellidan infested crayfish exhibited lower aggression levels than their uninfested counterparts, which is predicted to be costly in terms of fitness, given the naturally aggressive nature of these animals [56]. The poorer performance of infested animals during agnostic interactions suggests that branchiobdellidan infestation may reduce the host’s ability to access resources such as food, shelter and reproductive partners. The effects of branchiobdellidans on crayfish behaviour may be sex dependant with more pronounced effects noted for females. Regardless, branchiobdellidans do affect crayfish aggressiveness, which may alter development of dominance hierarchies, with potential consequences for host population dynamics.

As branchiobdellidans have a direct life cycle and are transmitted during host-host contact [57], reduced host aggression may result in decreased worm transmission rates. We, however found no evidence that the proportion of branchiobdellidans transmitted was correlated with the duration of contact between the infested and uninfested crayfish. Conversely, there was a significant positive correlation between infestation intensity (i.e., the number of worms on the infested crayfish at the start of the trial) and the proportion of worms transmitted to the originally uninfested host. Branchiobdellidan intensity may therefore be a better predictor of transmission rate than duration of host-host contact, although presumably both factors are crucial for worm transmission in wild populations.

In terms of foraging efficiency infested animals captured, on average, fewer prey items than their uninfested counterparts. Among infested crayfish, infestation intensity was negatively correlated with the number of prey caught. By decreasing foraging efficiency branchiobdellidans may reduce long term growth in the wild where prey is limited and more spatially distributed. This may carry high fitness costs for crayfish where size is correlated to dominance [56] and reproductive success [58, 59]. Further studies are needed to elucidate whether *X. victoriensis* infestation is detrimental to crayfish fitness under natural conditions. Such studies are vital if we are to predict the effects of *X. victoriensis* infestation on signal crayfish invasion.

## Conclusions

This is first report of branchiobdellidans affecting host behaviour, in this case competitive interactions and foraging of crayfish. The mechanism driving these behavioural changes is unclear, but we hypothesize that it may be driven by branchiobdellidans stimulating mechano-receptors on the crayfish exoskeleton, and thus causing interference with other behaviours (e.g., foraging and intraspecific interactions). A similar mechanism was recently proposed as being the cause of reduced foraging aptitude and predator detection in flea infested gerbilline rodents [48]. Regardless of the causal mechanism, these behavioural changes are likely to disrupt the social structure, and potentially growth rate and/or dispersal of branchiobdellidan infested signal crayfish populations in the wild. As crayfish are keystone species that interact with organisms on multiple trophic levels and alter nutrient cycling processes [21] such changes to signal crayfish population dynamics, as well as to individual animal behaviour, may have important ecosystem level consequences. This is particularly salient considering the widespread invasive range of these crayfish [16–19].
